# Supplementary material for: Population fraction of Parkinson’s disease attributable to preventable risk factors
Source: medRxiv. 2023 May 19:2023.05.19.23290231. Preprint. [Version 1] doi: 10.1101/2023.05.19.23290231 (PMC10246145; doi:10.1101/2023.05.19.23290231)
Supplement: Supplement 1 [file media-1.pdf]

Supplementary data. Full data used in study.

Sample ID	Case status	Sex	Age	PD oset age	RBD	MTBI/concussion	MTBI/concussion age	Repeated blows to head	Agent orange /chemical warfare exposure	Pesticide/herbicide exposure	Pesticide/herbicide exposure duration	State subject born	State current residence	Hispanic or Latino	Race	Jewish ancestry	Family history PD	Family history PD degree	Lost 10 lbs last year	Constipation
PAF0001																				
PAF0002																				
...																				
...																				
PAF1223																				

Data will be populated with peer-reviwed publication.

Supplementary Material / Software used in the study

Software or database name	Version	URL	RRIDs if applicable
PROGENY	9	http://www.progenygenetics.com/	RRID:SCR_006647
Microsoft Excel	16.0.15601.20526	https://www.microsoft.com/en-gb/	RRID:SCR_016137
R	4.1.3	https://cran.r-project.org/bin/windows/base/old/4.1.3/	RRID:SCR_001905
RStudio Connect	2023.03.0	https://docs.posit.co/previous-versions/connect/	RRID:SCR_000432
AF R Package	0.1.5	https://cran.r-project.org/package=AF	
data.table R Package	1.14.0	https://cran.r-project.org/package=data	
ggplot2 R Package	3.4.2	https://cran.r-project.org/package=ggplot2	
maps R Package	3.4.1	https://cran.r-project.org/package=maps	
mapsproj R Package	1.2.11	https://cran.r-project.org/package=mapsproj	
officer R Package	0.6.1	https://cran.r-project.org/package=officer	
openxlsx R Package	4.2.3	https://cran.r-project.org/package=openxlsx	
pairwiseCI R Package	0.1-27	https://cran.r-project.org/package=pairwiseCI	
renv R Package	0.13.2	https://cran.r-project.org/package=renv	
table1 R Package	1.4.3	https://cran.r-project.org/package=table	
targets R Package	0.13.1	https://cran.r-project.org/package=targets	
